# Novel prognostic biomarker TBC1D1 is associated with immunotherapy resistance in gliomas

**DOI:** 10.3389/fimmu.2024.1372113

**Published:** 2024-03-11

**Authors:** Daqiang Song, Qian Yang, Liuying Li, Yuxian Wei, Chong Zhang, Huimin Du, Guosheng Ren, Hongzhong Li

**Affiliations:** ^1^ Chongqing Key Laboratory of Molecular Oncology and Epigenetics, The First Affiliated Hospital of Chongqing Medical University, Chongqing, China; ^2^ Department of Pharmacy, Chongqing Medical University, Chongqing, China; ^3^ Clinical Molecular Medicine Testing Center, The First Affiliated Hospital of Chongqing Medical University, Chongqing, China; ^4^ Department of Breast and Thyroid Surgery, The First Affiliated Hospital of Chongqing Medical University, Chongqing, China; ^5^ Department of Ultrasound, The First Affiliated Hospital of Chongqing Medical University, Chongqing, China; ^6^ Department of Oncology, The First Affiliated Hospital of Chongqing Medical University, Chongqing, China

**Keywords:** TBC1D1, prognosis, biomarker, immunotherapy, gliomas

## Abstract

**Background:**

Glioma, an aggressive brain tumor, poses a challenge in understanding the mechanisms of treatment resistance, despite promising results from immunotherapy.

**Methods:**

We identified genes associated with immunotherapy resistance through an analysis of The Cancer Genome Atlas (TCGA), Chinese Glioma Genome Atlas (CGGA), and Gene Expression Omnibus (GEO) databases. Subsequently, qRT-PCR and western blot analyses were conducted to measure the mRNA and protein levels of TBC1 Domain Family Member 1 (TBC1D1), respectively. Additionally, Gene Set Enrichment Analysis (GSEA) was employed to reveal relevant signaling pathways, and the expression of TBC1D1 in immune cells was analyzed using single-cell RNA sequencing (scRNA-seq) data from GEO database. Tumor Immune Dysfunction and Exclusion (TIDE) database was utilized to assess T-cell function, while Tumor Immunotherapy Gene Expression Resource (TIGER) database was employed to evaluate immunotherapy resistance in relation to TBC1D1. Furthermore, the predictive performance of molecules on prognosis was assessed using Kaplan-Meier plots, nomograms, and ROC curves.

**Results:**

The levels of TBC1D1 were significantly elevated in tumor tissue from glioma patients. Furthermore, high TBC1D1 expression was observed in macrophages compared to other cells, which negatively impacted T cell function, impaired immunotherapy response, promoted treatment tolerance, and led to poor prognosis. Inhibition of TBC1D1 was found to potentially synergistically enhance the efficacy of immunotherapy and prolong the survival of cancer patients with gliomas.

**Conclusion:**

Heightened expression of TBC1D1 may facilitate an immunosuppressive microenvironment and predict a poor prognosis. Blocking TBC1D1 could minimize immunotherapy resistance in cancer patients with gliomas.

## Introduction

Glioma, renowned for its aggressive and malignant nature, has been the subject of extensive medical research due to its grim prognosis and limited treatment options. The current conventional modalities, encompassing radiotherapy, chemotherapy, and surgery, have exhibited restricted efficacy in treating glioma, emphasizing the urgent requirement for innovative therapeutic approaches (1, 2). Immunotherapy has emerged as a rapidly advancing modality, demonstrating significant progress in treating human cancers (3, 4). However, despite its promising results, immunotherapy resistance remains a significant hurdle in glioma treatment. Biomolecules play a pivotal role in the interaction between tumor cells and the immune system, influencing the sensitivity of tumor cells to immune attack by modulating surface markers, cytokines, and metabolites (5–7). Therefore, a comprehensive understanding of the functions and interactions of these biomolecules is essential to investigate the mechanisms of immunotherapy resistance. Strategies targeting these molecules may prove effective in overcoming immunotherapy resistance in glioma treatment.

TBC1 Domain Family Member 1 (TBC1D1) is involved in regulating various cellular processes, such as the cell cycle, apoptosis, cell migration, and cell differentiation, serving as a GTPase-activating protein according to prior research (8). Additionally, TBC1D1 has been identified as an important regulator in some cancers (9). However, its specific role and mechanism in glioma remain unclear. Despite its significance in other cancer types, limited knowledge exists regarding the function of TBC1D1 in glioma, necessitating further investigation. A detailed exploration of the function and mechanism of TBC1D1 in glioma is crucial to gain fresh insights and develop strategies for treating the disease.

The primary objective of this study is to comprehensively investigate the function and mechanism of TBC1D1 related to glioma’s immunotherapy resistance. To achieve this goal, we will employ a combination of bulk and single-cell sequencing analysis to identify the specific function of TBC1D1 in glioma immunotherapy. Specifically, our analysis will focus on the expression levels of TBC1D1 in glioma and its relevance to tumor immunotherapy response. Additionally, we will examine the impact of TBC1D1 on tumor immune evasion and drug resistance to uncover its mechanism in glioma. Our aim is to thoroughly elucidate the involvement of TBC1D1 in glioma’s resistance to immunotherapy and provide substantial support for the development of more effective immunotherapeutic approaches.

## Materials and methods

### Cell culture

The human HA1800 and, 1321N1 cell lines were acquired from the European Collection of Cell Culture. They were cultured in Dulbecco’s Modified Eagle Medium (DMEM) supplemented with 10% fetal bovine serum (04-001-1Acs, BI) and 1% streptomycin and penicillin (15140122, Gbico), and maintained at 37°C in a humidified atmosphere composed of 95% air and 5% CO2, with medium changes every other day.

### Real-time qPCR

RNA was extracted from the cells as described previously (10). The extracted RNA was then reverse-transcribed to cDNA using the RT Kit (HY-K0511A, MedChemExpress). Subsequently, the SuperReal PreMix Plus was used for PCR analysis and run in the Real-Time PCR System. We used Primer Premier 5.0 program to design the TBC1D1 primer. TBC1D1: Forward: 5’-CCTGCGCTACATCACACCC-3’ and reverse: 5’-CATGCGGTCTGGAACACTC-3’ and amplicon size was 175 bp. GAPDH: 5’-TGTGGGCATCAATGGATTTGG-3’ (forward) and 5’-ACACCATGTATTCCGGGTCAAT-3’ (reverse) and amplicon size was 116 bp (Sangon). The resulting data were normalized to GAPDH expression via the CFX Manager software (version 3.0), facilitating the evaluation of TBC1D1 mRNA expression levels.

### Western blot

Protein extraction was performed using RIPA lysis buffer (p0013B, Beyotime), followed by separation through 12% SDS-PAGE electrophoresis and transfer onto PVDF membranes. Thereafter, the membranes underwent blocking with 5% nonfat milk, succeeded by treatment utilizing either the TBC1D1 antibody (22124-1-AP, Proteintech) or the β-actin antibody (66009-1-Ig, Proteintech), and subsequently, on the following day, exposure to an HRP-linked anti-rabbit IgG antibody (BL003A, Biosharp). The visualization of the protein bands was attained using ECL reagents (34577, Thermo Fisher), and Image J software was employed to calculate the optical densities to determine TBC1D1 protein expression levels.

### Induction of different cell types

To acquire bone marrow cells, femurs were harvested from 8- to 10-week-old C57BL/6 mice (purchased from Ensiweier) and red blood cells were lysed using erythrocytes lysate (BL503A, Biosharp). Cell culture involved the use of complete DMEM medium (HyClone) with 20% L929 cell conditioned medium at 37°C and 5% CO2. The medium was refreshed on day 4 to obtain mature M0 macrophages by day 7, while M2 macrophages were induced by IL4 protein (1 ng/mL, HY-P70653, MedChemExpress), M1 macrophages by lipopolysaccharide stimulation, and MDSC differentiation was initiated by IL6 protein (5 ng/mL, HY-P7063, MedChemExpress). Single cell suspensions were obtained from spleens collected from wild-type C57BL/6 following erythrocyte lysis. T cell activation was achieved using anti-CD3 antibody (2.5 mg/mL, 145-2C11, BioLegend) and anti-CD28 antibody (3 mg/mL, 102102, BioLegend). Lastly, fibroblasts were isolated from tumors harvested from wild-type C57BL/6 mice, and the expression levels of TBC1D1 were confirmed in each of these cell types.

### Data acquisition

To explore the potential association between TBC1D1 expression and overall survival (OS), we retrieved data from several databases: CGGA (http://www.cgga.org.cn), TCGA (https://www.cancer.gov/ccg/research/genome-sequencing/tcga), GTEx (https://www.genome.gov/Funded-Programs-Projects/Genotype-Tissue-Expression-Project), and GEO (https://www.ncbi.nlm.nih.gov/geo). These databases provided access to expression matrices of the TBC1D1 gene as well as clinical information across normal (N = 1152) and tumor (N = 523) tissues. Prior to analysis, we standardized the data using R (version 4.3.0). Subsequently, we utilized the limma package to identify genes with differential expression linked to TBC1D1 expression, applying a threshold of |log2 FC| ≥ 1 and an adjusted *P*-value (FDR) of < 0.05.

### The human protein atlas

The Human Protein Atlas (HPA) database, established in Sweden in, 2003, endeavors to comprehensively map all human proteins within cells, tissues, and organs by employing a variety of omics technologies including antibody-based imaging, mass spectrometry-based proteomics, systems biology, and transcriptomics (11, 12). In this study, we leveraged the HPA database to validate the intracellular localization of the TBC1D1 protein, assess its mRNA expression in somatic cells, tissues, and immune cells, and compare its protein expression in normal and tumor tissues.

### LASSO analysis

LASSO analysis was conducted as previously described (10). In this study, we utilized LASSO analysis to build a prognostic model and identify genes associated with prognosis.

### Immune infiltration analysis

In this study, cancer patients were divided into two groups based on TBC1D1 expression. Subsequently, we employed CIBERSORT to assess the levels of immune cell infiltration in the tumor tissues.

### Tumor immune single-cell hub

Tumor Immune Single-cell (TISCH) database is an available resource facilitates the exploration of the tumor microenvironment (TME) via single-cell RNA sequencing (scRNA-seq) data (13, 14). We analyzed the distribution and expression of TBC1D1 in diverse cell types within the glioma microenvironment using the TISCH.

### GSEA analysis

We employed the GSEA computational method to assess the statistical significance of a preselected gene set (15–18). Following correlation analysis, we generated an initial list of gene categories. These categories were then segmented into various groups for each analysis, involving, 1000 permutations of gene sets to identify any disparities among them. The results of this analysis aided in identifying the critical genetic functions and signaling pathways linked to TBC1D1.

### Protein-protein interaction analysis

STRING database is dedicated to predicting protein-protein interactions (PPI) and employs computational predictions, cross-organism knowledge transfer, and curated data from other databases to identify both direct and indirect associations (19, 20). In this study, we utilized the STRING database to investigate protein-protein interactions.

### Tumor immune dysfunction and exclusion

Tumor immune dysfunction and exclusion (TIDE) tool is designed to predict transcriptomic biomarkers for immunotherapy response by analyzing the gene expression profile of a tumor before treatment (21–23). In this study, we used the TIDE to explore the association between TBC1D1 expression and T cell function in multiple human cancers, with a specific focus on glioma.

### Statistics

We analyzed the RNA-sequencing data using R (version 4.3.0). To compare the outcomes between the experimental and control groups, we employed a two-tailed Student’s t-test and visualized data by using GraphPad Prism (version 8.4.0). Furthermore, for multiple comparisons, we performed a one-way ANOVA (*P* < 0.05 = “*”, *P* < 0.01 = “**”, *P* < 0.001 = “***”, *P* < 0.0001 = “****”, P > 0.05 = ns).

## Results

### TBC1D1 has been identified as a significant marker associated with prognosis and therapy resistance

**Figure 1:**
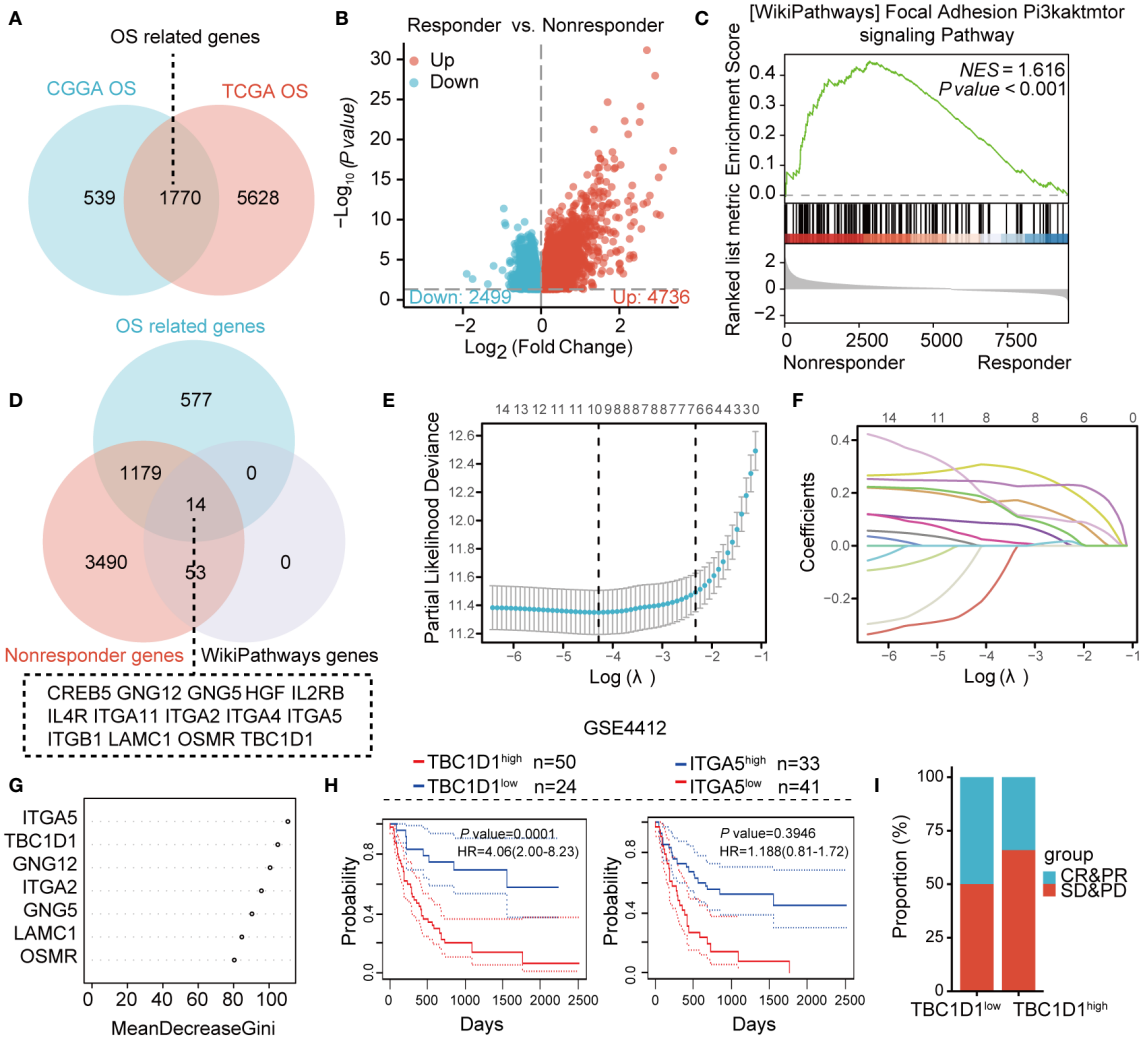
Integration of multiple datasets identifying markers correlated patient prognosis and immunotherapy resistance. **(A)** Venn diagram illustrating the related genes associated with overall survival from two separate datasets. **(B)** Volcano plot displays the differentially expressed genes in both responders and non-responders. **(C)** GSEA analysis demonstrating enriched signaling pathways. **(D)** Venn diagram illustrating the shared genes among three datasets. **(E, F)** Results of Lasso regression analysis for identifying prognosis-related genes. **(G)** Gene importance on the horizontal axis and the corresponding genes from **(F)** on the vertical axis. **(H)** Survival analysis for TBC1D1 and ITGA5 in cancer patients with glioma. **(I)** Effect of TBC1D1 on the therapy response of cancer patients with glioma.

Survival data analyses from CGGA and TCGA databases for glioma cancer patients revealed, 2309 and, 7398 differentially expressed genes, respectively. The intersection of these gene sets identified, 1770 genes linked to overall survival (OS) (**Figure 1A**). Among non-responders with drug therapy resistance, 4736 genes showed significant upregulation (**Figure 1B**). Further analysis of the upregulated genes for enrichment in signaling pathways using GSEA revealed a significant enrichment of the PI3K/AKT/mTOR pathway in the tumor tissue of non-responders (**Figure 1C**). The validation of core genes associated with patient prognosis and therapy resistance involved analyzing the common genes from three datasets: OS-related genes, up-regulated genes from non-responders, and those from the PI3K/AKT/mTOR signaling pathway. This analysis revealed fourteen core genes, which were further examined using LASSO regression, indicating that seven critical genes, including ITGA5, TBC1D1, GNG12, ITGA2, GNG5, LAMC1, and OSMR, were significant (**Figures 1D–F**). A random forest tree analysis confirmed the importance of ITGA5 and TBC1D1 as the most critical genes (**Figure 1G**). Interestingly, while ITGA5 showed no impact on patient survival, high expression of TBC1D1 was linked to an unfavorable prognosis (**Figure 1H**). Patients with high levels of TBC1D1 in their tumors exhibited poor therapy efficacy (**Figure 1I**). Overall, TBC1D1 emerged as a prognostic marker positively associated with therapy resistance.

### TBC1D1 was highly expressed in the multiple tumor tissues

**Figure 2:**
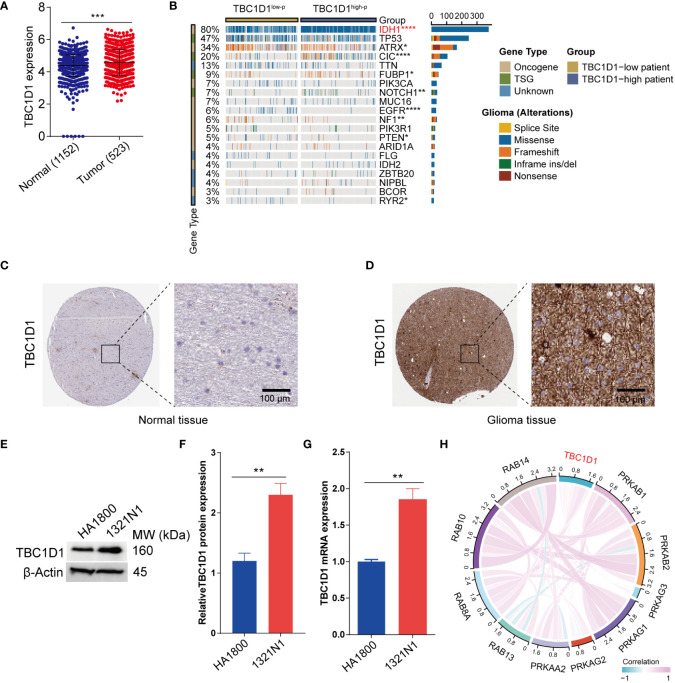
Expression of TBC1D1 in glioma and associated gene mutations. **(A)** Differential expression of TBC1D1 in normal and glioma tissues. **(B)** Waterfall plot showing the correlation between TBC1D1 and gene mutation frequency. **(C)** Expression levels of TBC1D1 in normal tissues from HPA database. **(D)** Expression levels of TBC1D1 in glioma tissues from HPA database. **(E)** Bands showing the expression of TBC1D1 protein in normal cells versus glioma cells. **(F)** Histogram visualization of protein expression differences. **(G)** Differential expression of TBC1D1 mRNA in normal versus glioma cells. **(H)** Interaction of TBC1D1 with core genes in glioma. *P* < 0.05 = “*”, *P* < 0.01 = “**”, *P* < 0.001 = “***”, *P* < 0.0001 = “****”, *P >*0.05 = ns.

Transcriptome sequencing data from the TCGA and GTEx databases were analyzed to evaluate TBC1D1 expression levels. The findings indicated a notable upregulation of TBC1D1 expression in glioma tissues compared to normal tissues (**Figure 2A**). This pattern was consistent across various human tumors, including ACC, CHOL, DLBC, HNSC, KICH, LAML, LIHC, MESO, OV, PCPG, TGCT, UCS, and UVM (**Supplementary Figure S1A**). Paired sample analysis also confirmed elevated TBC1D1 expression in tumor tissues (**Supplementary Figure S1B**). A recent genomewide mutational analysis of gliomas uncovered somatic mutations in the isocitrate dehydrogenase 1 (IDH1) gene in a subset of these tumors. Similarly, glioma patients with high-TBC1D1 tumors exhibited a higher frequency of mutations in IDH1 (**Figure 2B**). Immunohistochemical analysis validated higher TBC1D1 expression levels in tumor tissues, and a substantial increase in TBC1D1 expression was observed in the glioma cell line, 1321N1 compared to the normal cell line HA1800 (**Figures 2C–G**). In addition, the study identified the concurrent expression of 10 genes alongside TBC1D1 in gliomas, implying their potential contribution to tumor development (**Figure 2H**).

### TBC1D1 is associated with various clinicopathological variables of glioma

**Figure 3:**
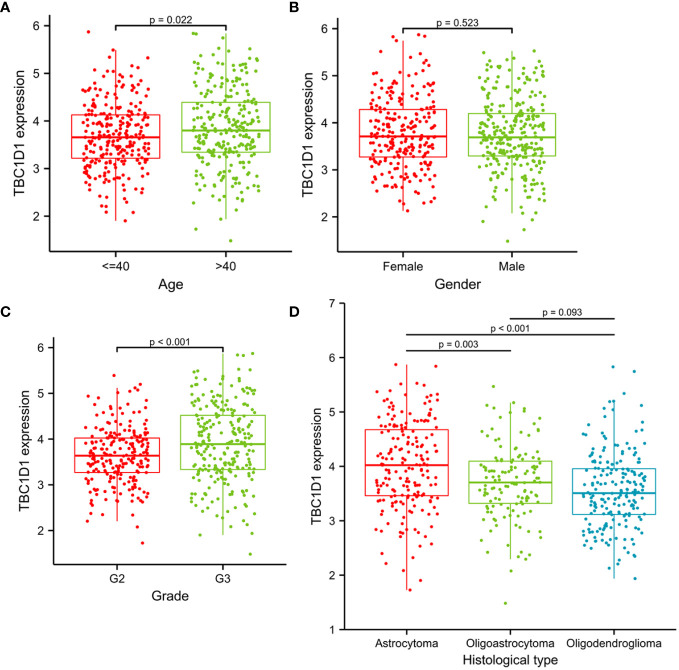
The associations between TBC1D1 and clinicopathological variables. **(A)** Associations between TBC1D1 expression and age. **(B)** Associations between TBC1D1 expression and gender. **(C)** Associations between TBC1D1 expression and grade. **(D)** Associations between TBC1D1 expression and histological type.

Logistic regression analysis was employed to investigate the relationship between TBC1D1 and various clinicopathological variables using a relevant clinical dataset. The findings revealed a notable elevation in TBC1D1 levels in glioma patients over 40 years old compared to those under 40 years old (**Figure 3A**). Additionally, a significant correlation between TBC1D1 expression and tumor grade was observed, with an increase in expression as the tumor grade advanced from 2 to 3 (**Figure 3C**). Remarkably, varying levels of TBC1D1 were observed in different histological types, with higher expression in astrocytomas compared to oligoastrocytomas or oligodendrogliomas (**Figure 3D**). In contrast, gender showed less significance as a clinical variable (**Figure 3B**). In conclusion, the results suggest a notable correlation between TBC1D1 expression and key clinical variables such as patient age, tumor grade, and histological type.

### Constructed nomogram on the basis of TBC1D1 predicts patient prognosis

**Figure 4:**
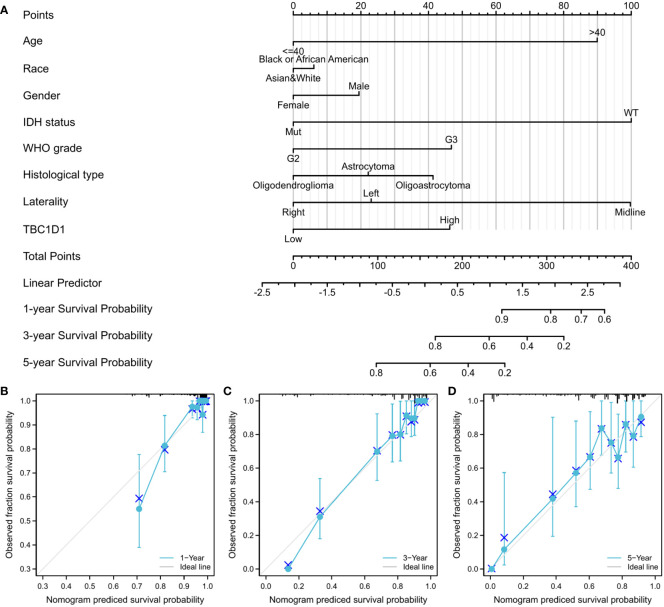
TBC1D1 based nomogram and relevant evaluation. **(A)** Nomogram according to eight clinicopathological factors, including TBC1D1, age, race, gender, grade, IDH1 status, histological type and laterality. **(B)** Calibration curve at 1 year. **(C)** Calibration curve at 3 years. **(D)** Calibration curve at 5 years.

The developed nomogram revealed a robust association between TBC1D1 and various clinical variables (**Figure 4A**). Specifically, the nomogram demonstrated exceptional predictive accuracy at three distinct time points, with C-indexes and AUC values of 0.817, 0.901, 0.897, and 0.803, respectively. Moreover, the calibration curves for 1-year, 3-year, and 5-year predictions displayed a high level of concordance between the predicted and actual outcomes (**Figures 4B–D**). These findings further validate the dependable performance of the TBC1D1-based nomogram in accurately predicting the prognosis of glioma patients.

### TBC1D1 is found to participate in various signaling pathways

**Figure 5:**
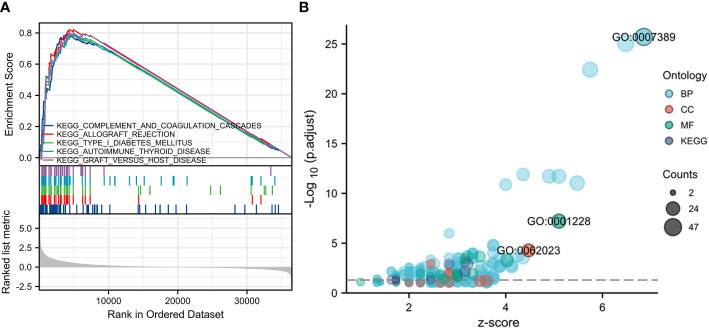
GSEA and GO analysis based on TBC1D1 expression in glioma. **(A)** Significant enrichment to five vital signaling pathways. **(B)** The genic functions involved in TBC1D1 in glioma.

Our GSEA analysis revealed strong evidence of a significant correlation between elevated expression of TBC1D1 and pathways related to type I diabetes mellitus, complement and coagulation cascades, allograft rejection, autoimmune thyroid disease, and graft versus host disease signaling (**Figure 5A**). Additionally, gene ontology (GO) analysis highlighted TBC1D1’s primary associations with pattern specification processes in biological functions and the extracellular matrix containing collagen in cellular components. Notably, TBC1D1’s molecular function was significantly linked to DNA-binding transcriptional activator activity and specificity for RNA polymerase II (**Figure 5B**). These findings emphasize the critical role of TBC1D1 in regulating these essential signaling pathways and gene functions in tumor progression.

### TBC1D1 exhibits increased expression in M2-like macrophages located within the glioma TME

**Figure 6:**
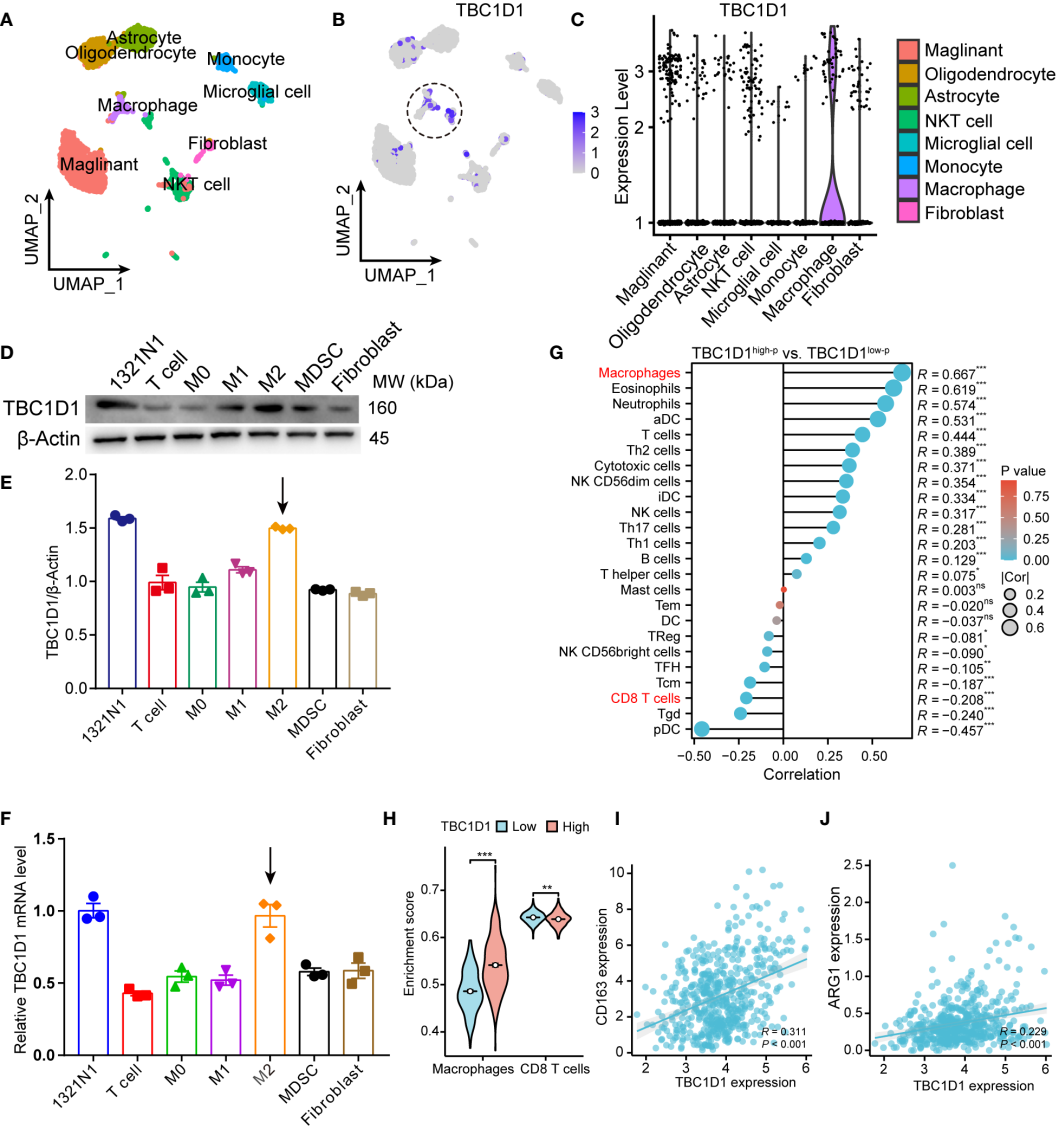
TBC1D1 expression in immune cells. **(A)** UMAP demonstrates the major immune cell populations in the TME of glioma (GSE141982). **(B)** UMAP demonstrates the distribution of TBC1D1 expression in various immune cell populations. **(C)** Violin diagram demonstrating the differential expression of TBC1D1 in different immune cell populations. **(D)** Protein expression of TBC1D1 in tumor cells and various immune cells. **(E)** Comparison of protein expression of TBC1D1 in tumor cells and various immune cells. **(F)** Differential mRNA expression of TBC1D1 in tumor cells and various immune cells. **(G)** Correlation analysis of TBC1D1 with immune cell infiltration. **(H)** Correlation of TBC1D1 with macrophages, T-cell enrichment fraction. **(I)** Correlation between TBC1D1 and CD163 expression. **(J)** Correlation between TBC1D1 and ARG1 expression. *P* < 0.05 = “*”, *P* < 0.01 = “**”, *P* < 0.001 = “***”, *P >*0.05 = ns.

In our investigation of the relationship between TBC1D1 and the TME, scRNA-seq data from glioma obtained from the GEO database were analyzed. The results revealed high expression of TBC1D1 in immune cells, particularly in macrophage populations (**Figures 6A–C**). Subsequent analysis of macrophage subpopulations showed predominant expression of TBC1D1 in the M2 subpopulation (**Supplementary Figures S2A–I**). Expanding our inquiry, we induced the differentiation of bone marrow-derived monocytes into different subtypes of macrophages and examined the protein and mRNA levels of TBC1D1 in cancer-associated fibroblasts and splenic T cells. Our findings indicated that TBC1D1 was expressed in M2-like macrophages, akin to tumor cells (**Figures 6D–F**). Stratifying glioma patients into two groups based on their TBC1D1 levels, it was observed that patients with elevated TBC1D1 levels exhibited high macrophage infiltration within the TME, while a noticeable negative correlation was observed between TBC1D1 and T cell infiltration levels (**Figures 6G, H**). Importantly, this trend was also observed in other types of gliomas, including glioblastoma multiforme and brain low-grade glioma (**Supplementary Figures S3A, B**). Additionally, TBC1D1 displayed a positive correlation with immunosuppressive macrophage markers such as CD163 and ARG1 (**Figures 6I, J**), suggesting that TBC1D1-positive macrophages could function as an immunosuppressive cell population, facilitating the establishment of an immunosuppressive TME and promoting tumor immune escape, ultimately accelerating tumorigenesis and progression.

### TBC1D1 reduces the effectiveness of immunotherapy and shows great accuracy in predicting patient prognosis

**Figure 7:**
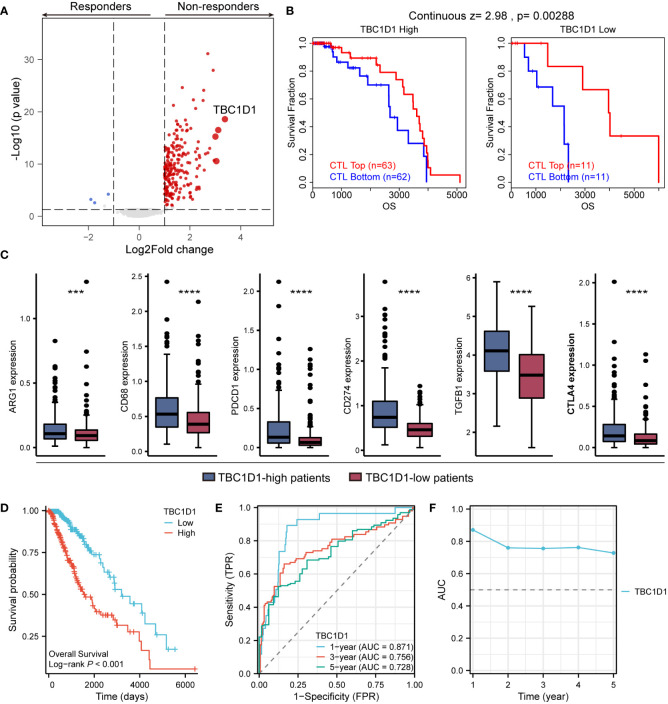
Effect of TBC1D1 on treatment outcome and prognosis in glioma patients. **(A)** Volcano plot demonstrating differentially expressed genes in glioma treatment responders versus non-responders. **(B)** TBC1D1 affects survival of glioma patients by regulating CTL function. **(C)** Expression of immunosuppression-related genes, including ARG1, CD68, PDCD1, CD274, TGFB1, CTLA4, in tumors of patients with high- and low-TBC1D1 expression. **(D)** Effect of TBC1D1 on survival of glioma patients. **(E)** ROC curves to evaluate the predictive ability of TBC1D1 on survival time of patients. **(F)** AUC curves to evaluate the efficacy of TBC1D1 in predicting patient survival. *P* < 0.05 = “*”, *P* < 0.01 = “**”, *P* < 0.001 = “***”, *P* < 0.0001 = “****”, *P* > 0.05 = ns.

An analysis of treatment outcomes in glioma patients from the TCGA database revealed a strong association between high TBC1D1 expression and non-responsive patients (**Figure 7A**). We further investigated the impact of TBC1D1 on treatment tolerance, focusing on its effect on the function of cytotoxic T lymphocytes (CTL), which play a crucial role in eliminating tumor cells. Our findings clearly indicated that high TBC1D1 expression impairs favorable prognosis in the context of high CTL function in tumor tissues, leading to poor survival outcomes among glioma patients. Conversely, low TBC1D1 expression was positively correlated with enhanced CTL function and improved patient survival (**Figure 7B**). This trend was observed in breast, colon, endometrial, melanoma, myeloma, and ovarian cancers (**Supplementary Figures S4A–F**). Our mechanistic study revealed that TBC1D1-mediated inhibition of CTL function could be attributed to the upregulation of various immune checkpoint molecules, including ARG1, CD68, PDCD1, CD274, TGFB1, and CTLA4, collectively impeding the anti-tumor immune response (**Figure 7C**). In animal models receiving immunotherapy in the TISMO database, high TBC1D1 expression significantly hindered the effectiveness of immune checkpoint blockade (ICB) treatment, resulting in an increase in non-responders (**Supplementary Figure S5A**). Analysis of the TIGER database of patients receiving anti-PD-1 immunotherapy for melanoma revealed that TBC1D1 impedes the effectiveness of the treatment, leading to suboptimal patient survival rates (**Supplementary Figure S5B**). Additionally, patients with high TBC1D1 expression exhibited increased infiltration of M2 tumor-associated macrophages (TAMs), showing significant elevation in markers including CD68 and ARG1 (**Figure 7C**). The M2 TAMs signature is a significant risk factor in patients with melanoma treated with immunotherapy (**Supplementary Figure S6A**), and patients with tumors exhibiting high M2 TAMs signature expression had a poor survival probability (**Supplementary Figure S6B**). Therefore, we posited that TBC1D1-positive macrophages could inhibit the effectiveness of immunotherapy in glioma patients, leading to immunotherapy tolerance and poor prognosis. Furthermore, we demonstrated the prognostic value of TBC1D1 using Kaplan-Meier curves (**Figure 7D**) and ROC curves and AUC values at different time points (**Figures 7E, F**). Overall, the study results indicated that macrophages with elevated TBC1D1 levels may play a crucial role in immunotherapy resistance and can act as a compelling biomarker when predicting the chances of survival for patients with gliomas.

## Discussion

In recent years, rapid advancements in bioinformatics have greatly enhanced disease diagnosis and prognosis (24, 25). In this study, bioinformatics analysis was employed to identify TBC1D1 as a potential biomarker for the prognosis and immunotherapy of cancer patients with gliomas. The TBC1D1 (Tre-2/Bub2/Cdc16 Structural Domain Family Member 1) gene encodes a protein that plays a crucial role in regulating several intracellular vesicle trafficking and membrane trafficking pathways, including endocytosis, autophagy, and insulin signaling (26, 27). Additionally, the TBC1D1 protein is involved in regulating glucose uptake and maintaining glucose homeostasis in muscle cells (28, 29). Despite its association with the progression and development of certain cancers, the precise biological functions and role of TBC1D1 in disease pathogenesis remain incompletely understood, underscoring the necessity for further research to identify it as a potential therapeutic target. In the context of glioma, despite its expression in numerous human cancers, previous research has paid little attention to the role of TBC1D1. To address this gap, we analyzed transcriptome data from various databases such as TCGA, GTEx, and GEO to explore the potential impact of TBC1D1 in glioma.

The poor prognosis and tumor recurrence in cancer patients are regulated by various factors, including changes in the migration ability of tumor cells (30). Therefore, it is crucial to identify these potential regulatory factors. In this study, the TBC1D1 gene exhibits elevated expression levels in tumor tissues and cells, particularly in glioma. Furthermore, this expression is significantly and positively associated with IDH1 gene mutations, which have been linked to a more unfavorable prognosis in glioma patients. Our hypothesis suggests that TBC1D1 may enhance the frequency of IDH1 mutations, ultimately influencing patient prognosis. Additionally, TBC1D1’s impact on glioma likely involves a complex interplay of multiple clinically relevant factors. Moreover, the construction of a survival probability prediction nomogram utilizing TBC1D1 expression at different time points demonstrated its efficacy as a reliable prognostic indicator.

Through GSEA and GO analysis, we gained valuable insights into the underlying mechanisms of TBC1D1. Our analysis revealed a notable enrichment of five critical signaling pathways and three gene functions in the high TBC1D1 expression group, highlighting their significance in mediating the effects of TBC1D1. This observation further supports the hypothesis that TBC1D1’s involvement in tumor progression encompasses the regulation of multiple signaling pathways, including those related to autoimmune thyroid disease. The immune system plays a crucial role in cancer development (31). To advance our comprehension of the impact of TBC1D1 on the TME, we conducted transcriptome sequencing analysis on single cells obtained from glioma patients in the GEO database. Our findings revealed notably higher expression of TBC1D1 in macrophages, contrasting with lower expression in other immune cell populations. The result was subsequently validated through experiments. Furthermore, TBC1D1 exhibited a positive correlation with macrophage infiltration and markers, while displaying a negative correlation with CD8^+^ T cells, an immune cell type renowned for its anti-tumor properties. Tumor-associated macrophages are pivotal contributors to the development of immunosuppressive microenvironments, capable of modulating the immune response to tumors. In contrast, CD8^+^ T cells play a crucial role in promoting tumor immunity by displaying potent anti-tumor activity (32–34). Therefore, based on these findings, we hypothesize that high-TBC1D1 macrophages within the TME may promote an immunosuppressive state, impairing CD8^+^ T cell function and enabling tumor immune escape, ultimately propelling tumor progression.

Our research also revealed that heightened TBC1D1 expression is closely linked to treatment resistance, compromising the effectiveness of immunotherapy and resulting in poor prognosis for glioma patients. Additionally, elevated TBC1D1 expression upregulates immune checkpoint molecules within tumor tissue, further contributing to immunotherapy resistance.

## Conclusion

Our research has revealed a significant positive correlation between TBC1D1 and IDH1 mutations in glioma patients, emphasizing the crucial involvement of TBC1D1 in modulating these mutations. Additionally, the presence of high-TBC1D1 macrophages contributes to the creation of an immunosuppressive TME, ultimately impacting the effectiveness of antitumor immunotherapy and resulting in treatment resistance. We hypothesize that by targeting TBC1D1 in combination with ICB, the efficacy of antitumor immunotherapy can be enhanced, potentially inhibiting tumor progression and improving patient survival.

## Data availability statement

The original contributions presented in the study are included in the article/**Supplementary Material**. Further inquiries can be directed to the corresponding authors.

## Ethics statement

The animal studies were approved by Ethics Committee of the First Affiliated Hospital of Chongqing Medical University 审批编号; 2023年科研伦理 (2023-6S). The studies were conducted in accordance with the local legislation and institutional requirements. Written informed consent was obtained from the owners for the participation of their animals in this study.

## Author contributions

DS: Writing – original draft, Writing – review & editing. QY: Writing – review & editing, Data curation, Formal analysis. LL: Writing – review & editing, Software. YW: Writing – review & editing, Formal analysis, Validation. CZ: Writing – review & editing, Investigation. HD: Writing – review & editing, Formal analysis. GR: Supervision, Writing – original draft. HL: Supervision, Writing – original draft, Funding acquisition, Visualization.

## Supplementary material

Supplementary Figure 1 The expression of TBC1D1 varies in human tumors. **(A)** In unpaired samples, the expression of TBC1D1 between normal and tumor tissues. **(B)** In paired samples, the expression of TBC1D1 between normal and tumor tissues. *P* < 0.05 = “*”, *P* < 0.01 = “**”, *P* < 0.001 = “***”, *P* > 0.05 = ns.

Supplementary Figure 2 The distribution and expression of TBC1D1 in glioma TME. **(A)** Cell populations in malignant tumor cell types. **(B, C)** Expression of TBC1D1 in different cell populations. **(D)** Cell populations in the main lineage cell types. **(E, F)** Expression of TBC1D1 in different cell populations. **(G)** Cell populations in minor lineage cell types. **(H, I)** Expression of TBC1D1 in different cell populations.

Supplementary Figure 3 The association between TBC1D1 and immune cell infiltration in patients with different types of gliomas. **(A)** Correlation between TBC1D1 and immune cell infiltration in low-grade gliomas of the brain. **(B)** Correlation between TBC1D1 and immune cell infiltration in glioblastoma multiforme. *P* < 0.05 = “*”, *P* < 0.01 = “**”, *P* < 0.001 = “***”, *P* > 0.05 = ns.

Supplementary Figure 4 Survival analysis of cancer patients. **(A–F)** The survival of cancer patients with high- and low-CTL infiltration levels in high- or low-TBC1D1 human tumors, including breast cancer **(A)**, colorectal cancer **(B)**, endometrial cancer **(C)**, melanoma **(D)**, myeloma **(E)**, and ovarian cancer **(F)**.

Supplementary Figure 5 Effect of the TBC1D1 on efficacy of ICB treatment. **(A)** Effect of TBC1D1 on the efficacy of immunotherapy in mice model. **(B)** Effect of TBC1D1 on the survival of melanoma patients undergoing anti-PD-1 immunotherapy.

Supplementary Figure 6 Effect of TBC1D1 on the effectiveness of M2 TAM-mediated immunotherapy. **(A)** Analysis of melanoma patients undergoing anti-PD-1 immunotherapy with TBC1D1-regulated M2 signature as a significant risk factor. **(B)** Effect of the TBC1D1-mediated M2 signature on patient survival.
