# Supplementary figures and images for: Novel prognostic biomarker TBC1D1 is associated with immunotherapy resistance in gliomas
Source: Front Immunol. 2024 Mar 11;15:1372113. doi: 10.3389/fimmu.2024.1372113 (PMC10961388; doi:10.3389/fimmu.2024.1372113)

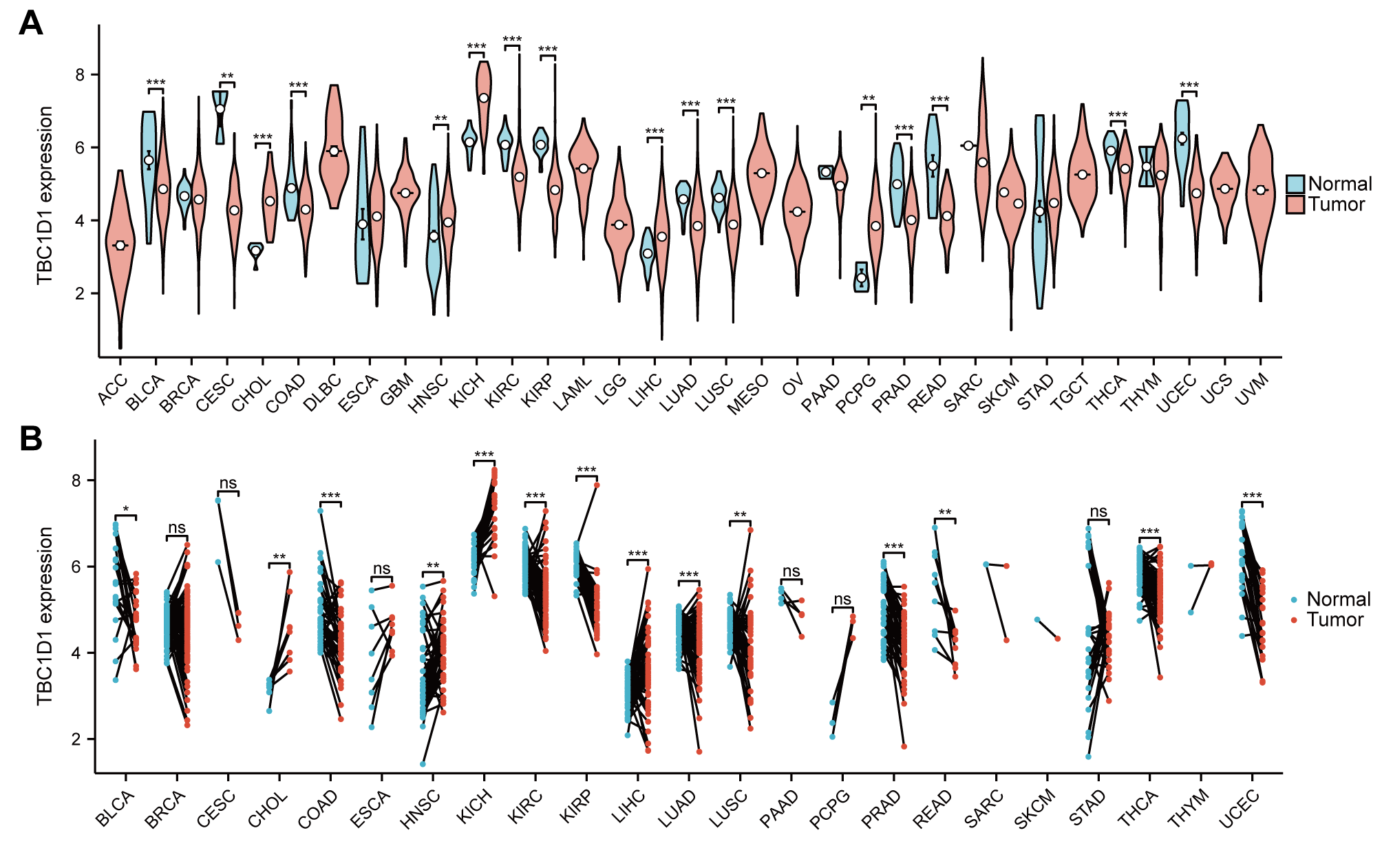

Supplement: Supplementary Figure 1 — The expression of TBC1D1 varies in human tumors. (A) In unpaired samples, the expression of TBC1D1 between normal and tumor tissues. (B) In paired samples, the expression of TBC1D1 between normal and tumor tissues. P < 0.05 = “*”, P < 0.01 = “**”, P < 0.001 = “***”, P > 0.05 = ns. [file Image_1.tif]

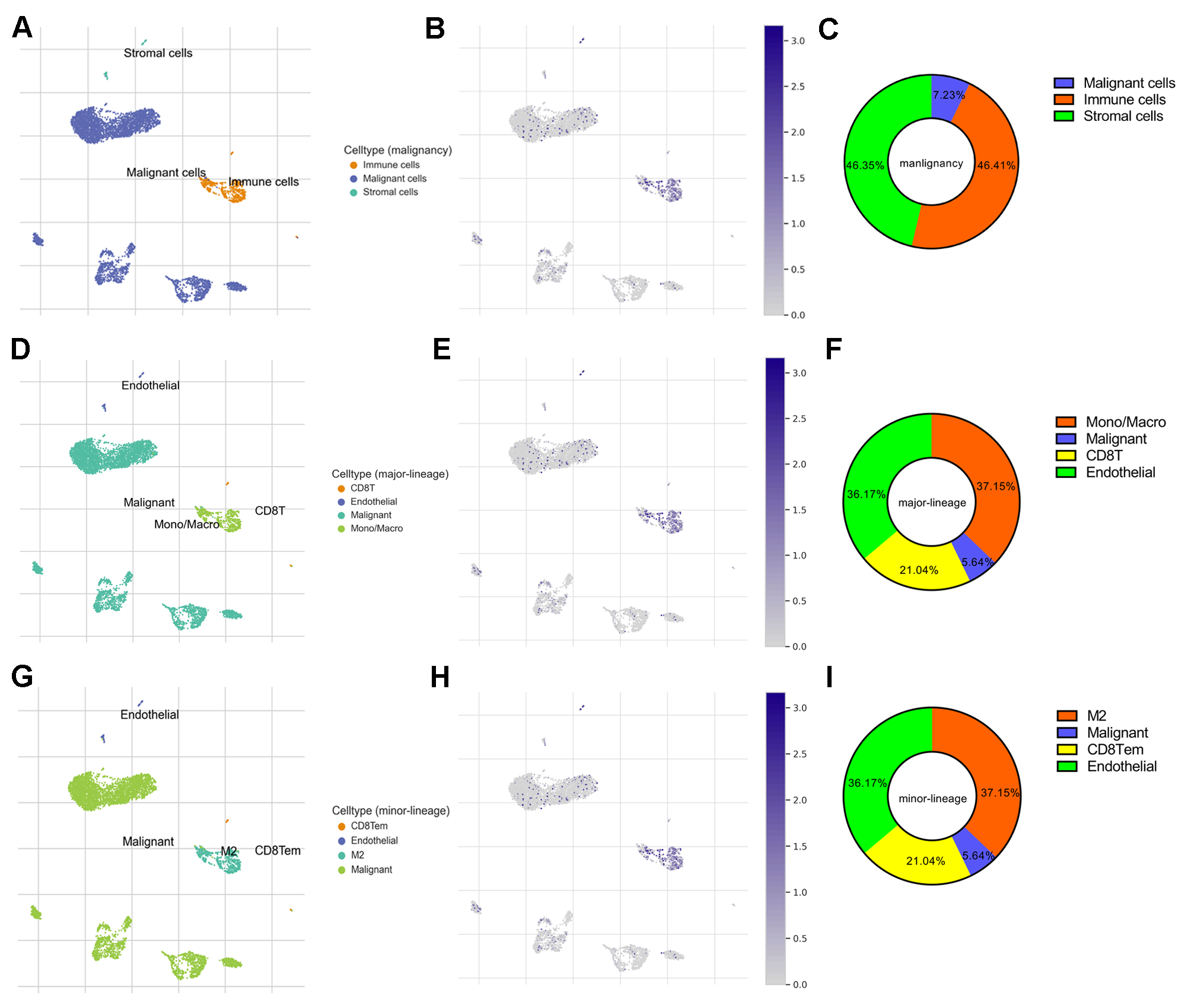

Supplement: Supplementary Figure 2 — The distribution and expression of TBC1D1 in glioma TME. (A) Cell populations in malignant tumor cell types. (B, C) Expression of TBC1D1 in different cell populations. (D) Cell populations in the main lineage cell types. (E, F) Expression of TBC1D1 in different cell populations. (G) Cell populations in minor lineage cell types. (H, I) Expression of TBC1D1 in different cell populations. [file Image_2.tif]

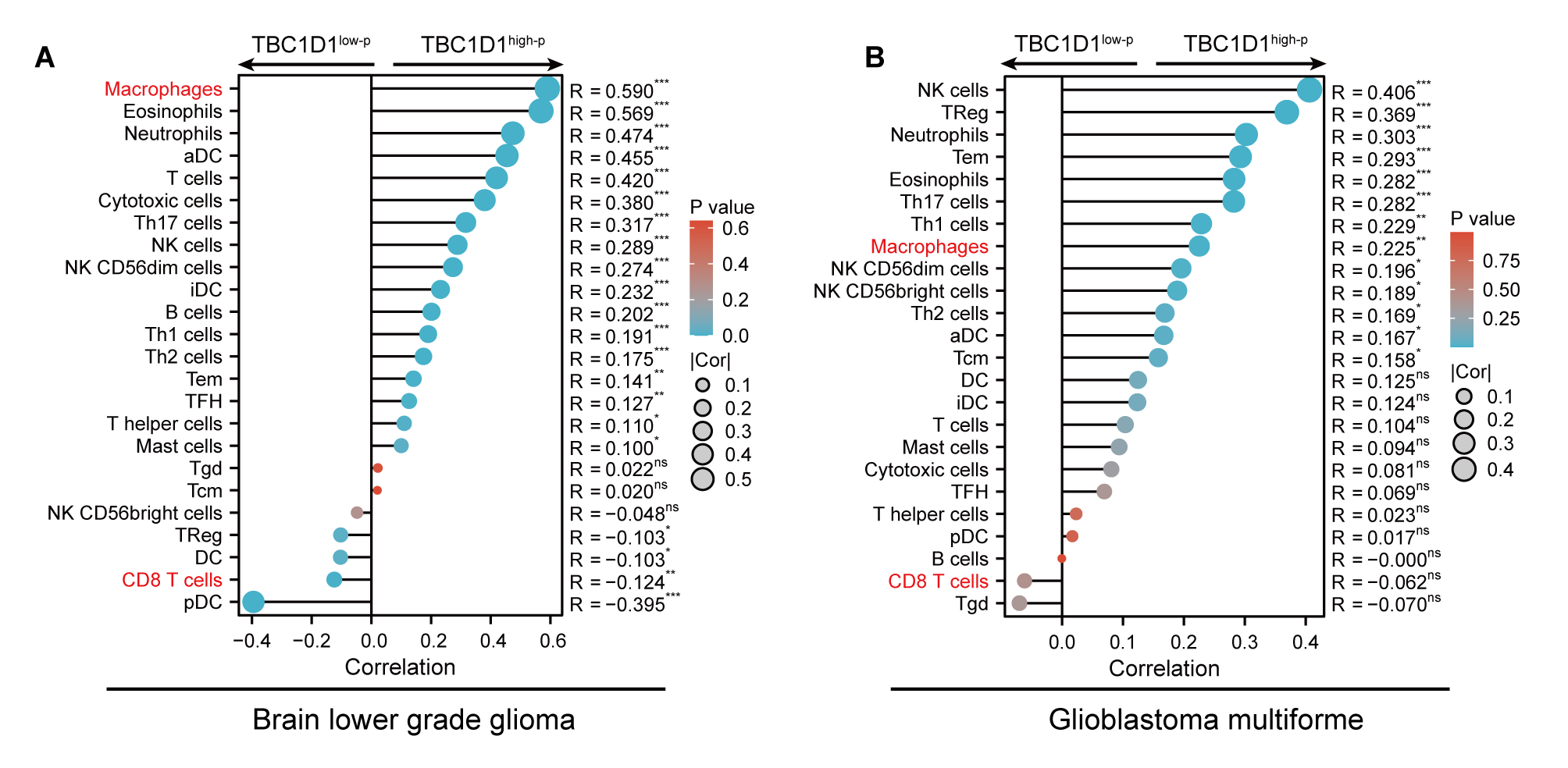

Supplement: Supplementary Figure 3 — The association between TBC1D1 and immune cell infiltration in patients with different types of gliomas. (A) Correlation between TBC1D1 and immune cell infiltration in low-grade gliomas of the brain. (B) Correlation between TBC1D1 and immune cell infiltration in glioblastoma multiforme. P < 0.05 = “*”, P < 0.01 = “**”, P < 0.001 = “***”, P > 0.05 = ns. [file Image_3.tif]

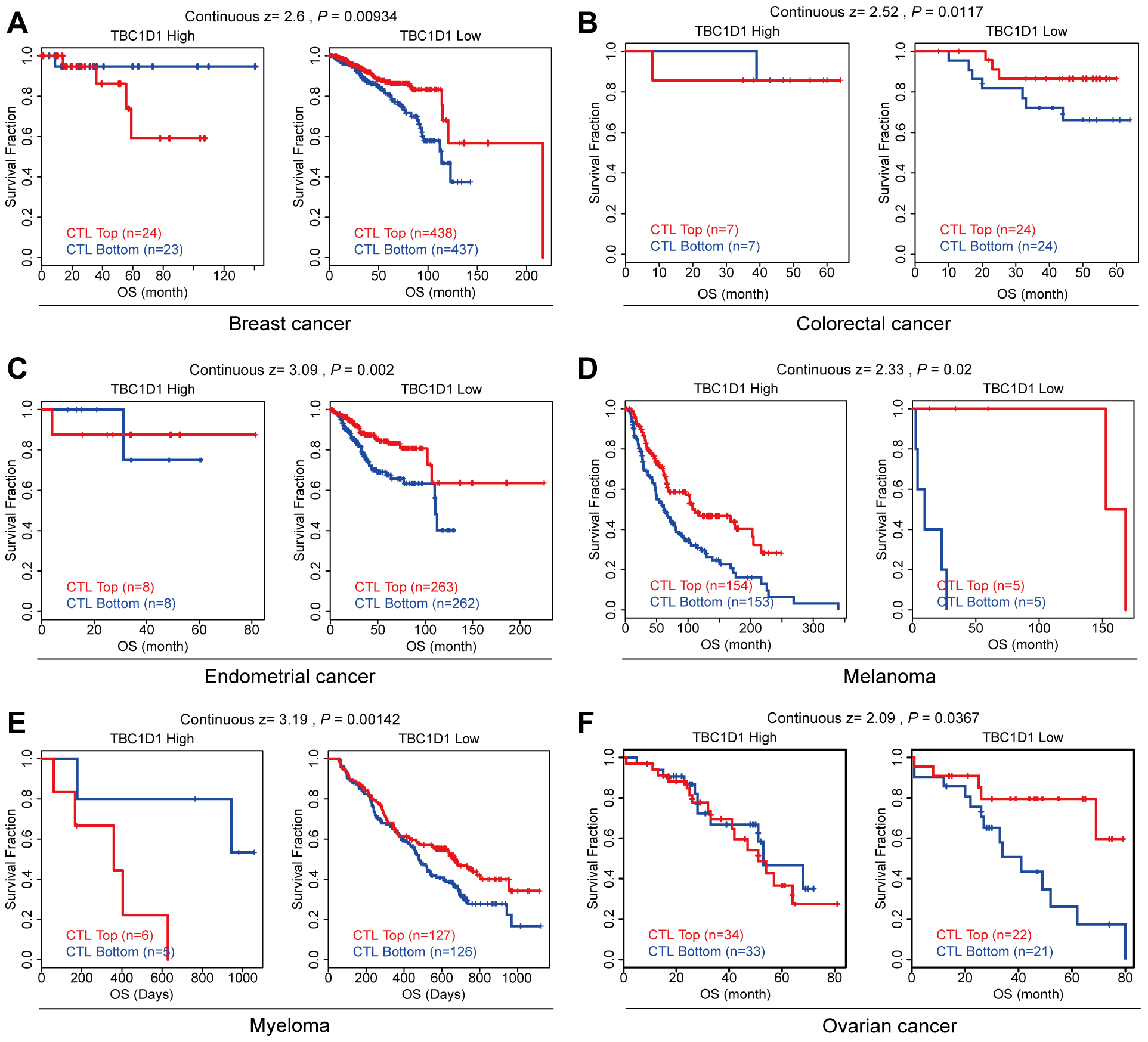

Supplement: Supplementary Figure 4 — Survival analysis of cancer patients. (A–F) The survival of cancer patients with high- and low-CTL infiltration levels in high- or low-TBC1D1 human tumors, including breast cancer (A), colorectal cancer (B), endometrial cancer (C), melanoma (D), myeloma (E), and ovarian cancer (F). [file Image_4.tif]

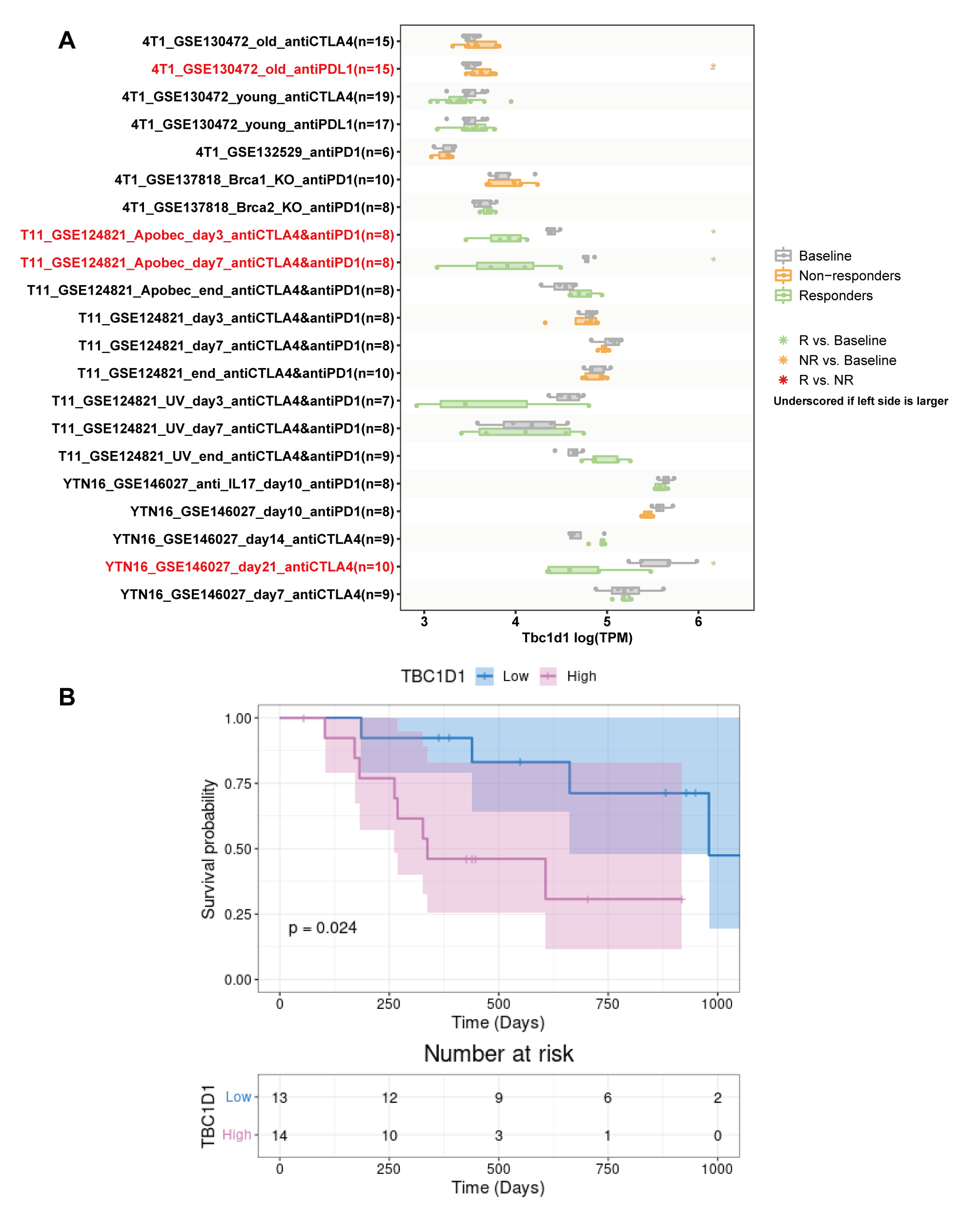

Supplement: Supplementary Figure 5 — Effect of the TBC1D1 on efficacy of ICB treatment. (A) Effect of TBC1D1 on the efficacy of immunotherapy in mice model. (B) Effect of TBC1D1 on the survival of melanoma patients undergoing anti-PD-1 immunotherapy. [file Image_5.tif]

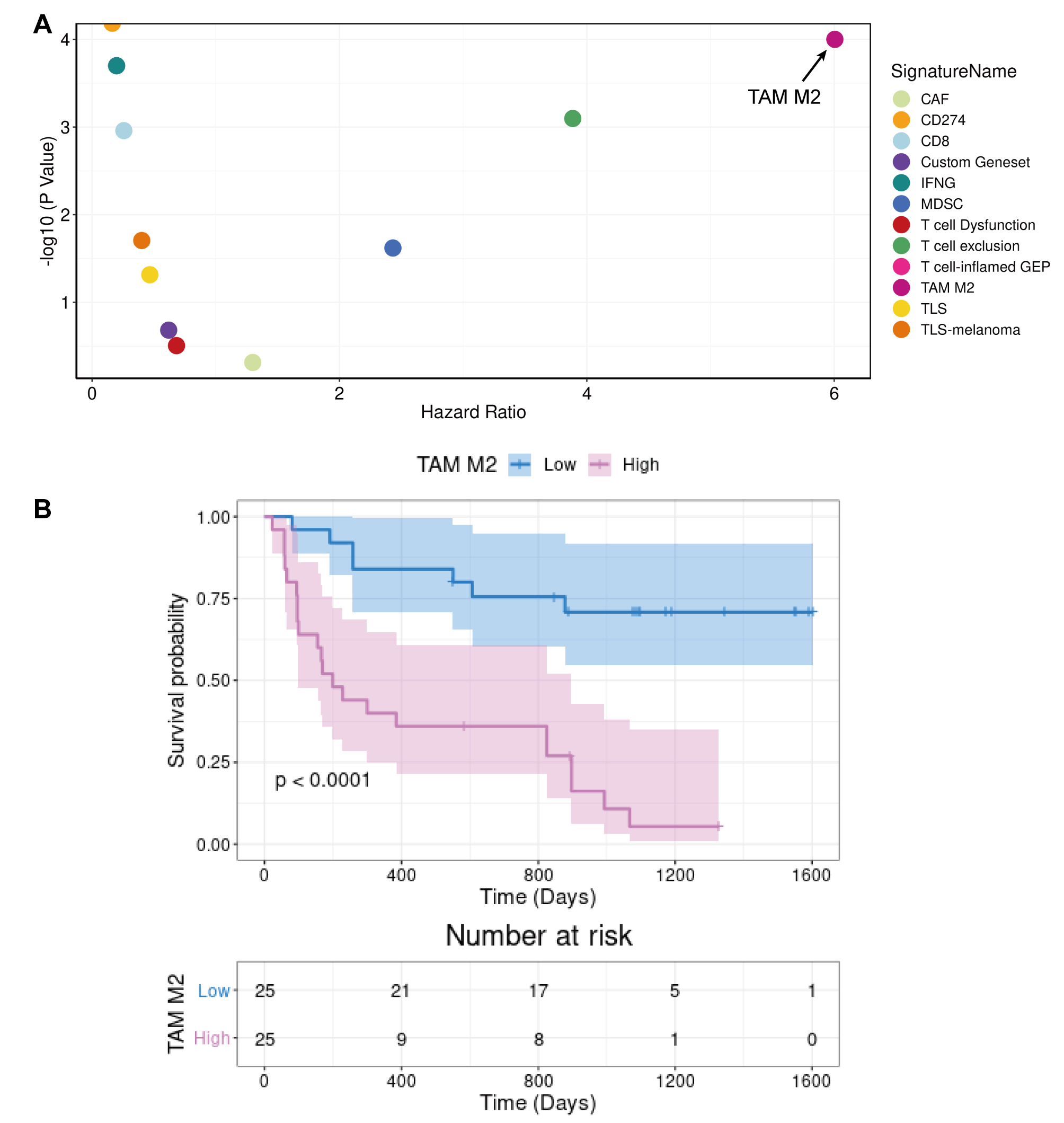

Supplement: Supplementary Figure 6 — Effect of TBC1D1 on the effectiveness of M2 TAM-mediated immunotherapy. (A) Analysis of melanoma patients undergoing anti-PD-1 immunotherapy with TBC1D1-regulated M2 signature as a significant risk factor. (B) Effect of the TBC1D1-mediated M2 signature on patient survival. [file Image_6.tif]
